# Cocrystal Prediction of Bexarotene by Graph Convolution Network and Bioavailability Improvement

**DOI:** 10.3390/pharmaceutics14102198

**Published:** 2022-10-16

**Authors:** Fu Xiao, Yinxiang Cheng, Jian-Rong Wang, Dingyan Wang, Yuanyuan Zhang, Kaixian Chen, Xuefeng Mei, Xiaomin Luo

**Affiliations:** 1 State Key Laboratory of Drug Research and Drug Discovery and Design Center, Pharmaceutical Analytical & Solid-State Chemistry Research Center, Shanghai Institute of Materia Medica, Chinese Academy of Sciences, Shanghai 201203, China; 2 School of Chinese Materia Medica, Nanjing University of Chinese Medicine, Nanjing 210023, China; 3 University of Chinese Academy of Sciences, Beijing 100049, China

**Keywords:** bexarotene, cocrystal prediction, GCN, bioavailability

## Abstract

Bexarotene (BEX) was approved by the FDA in 1999 for the treatment of cutaneous T-cell lymphoma (CTCL). The poor aqueous solubility causes the low bioavailability of the drug and thereby limits the clinical application. In this study, we developed a GCN-based deep learning model (CocrystalGCN) for in-silico screening of the cocrystals of BEX. The results show that our model obtained high performance relative to baseline models. The top 30 of 109 coformer candidates were scored by CocrystalGCN and then validated experimentally. Finally, cocrystals of BEX-pyrazine, BEX-2,5-dimethylpyrazine, BEX-methyl isonicotinate, and BEX-ethyl isonicotinate were successfully obtained. The crystal structures were determined by single-crystal X-ray diffraction. Powder X-ray diffraction, differential scanning calorimetry, and thermogravimetric analysis were utilized to characterize these multi-component forms. All cocrystals present superior solubility and dissolution over the parent drug. The pharmacokinetic studies show that the plasma exposures (AUC_0−8h_) of BEX-pyrazine and BEX-2,5-dimethylpyrazine are 1.7 and 1.8 times that of the commercially available BEX powder, respectively. This work sets a good example for integrating virtual prediction and experimental screening to discover the new cocrystals of water-insoluble drugs.

## 1. Introduction

BEX (Targretin ^®^) was approved by the FDA in 1999 for the treatment of cutaneous T-cell lymphoma (CTCL) [1] and is a member of the rexinoids family. As a selective agonist of the retinoid X receptor, it induces cell differentiation and apoptosis and is effective as an oral treatment for mycosis fungoides and Sézary syndrome [2], as documented in the national guidelines of the UK [3]. The clinic dose of BEX is 300 mg/m^2^ once daily, and the main toxicities of BEX include xeroderma, central hypothyroidism, and elevation of cholesterol, which can be managed with dose attenuation [4]. The clinical utilization of BEX was limited by the poor aqueous solubility, which led to malabsorption with an absolute bioavailability of less than 20% [5]. In order to increase the absorption and potentially reduce the dose, new solid forms of BEX with higher bioavailability should be developed.

Cocrystals are multicomponent crystals of fixed stoichiometry that contain different interactions such as hydrogen bonds, halogen bonds, π-π stacking, and van der Waals interactions [6]. Supramolecular synthons play a crucial role in cocrystal design and assembly. The hydrogen bonding between unlike functional groups is called heterosynthon, which can often be served as a structural design unit for cocrystal synthesis [7]. As a new type of crystalline material, pharmaceutical cocrystals possess the potential to alter the physical and chemical properties of active pharmaceutical ingredients (API), such as melting point [8], chemical stability [9,10], solubility [11,12], bioavailability [13,14], and so on. 

Traditional cocrystal screening methods such as solution crystallization [15], liquid-assisted grinding [16], dry grinding [17], and anti-solvent addition [18] are generally time-consuming, labor-intensive, and expensive economically. Thus, various computational approaches have been developed for in-silico cocrystal former (coformer) screening. Due to the abundant cocrystal data in the Cambridge Structural Database (CSD) [19], a series of knowledge-based methods including hydrogen bonding propensity [20,21], hydrogen bond motif searches [22,23], and molecular complementarity [24] are proposed. In addition, network-based methods [25,26] and ab initio methods such as molecular electrostatic potential surfaces (MEPS) [27,28], conductor-like screening models for real solvents (COSMO-RS) [29,30] are also available for cocrystal prediction.

With the development of computer science and the accumulation of data, machine learning-based approaches have been rapidly and widely applied in polymorphism and cocrystal prediction, such as crystal property prediction [31,32,33], crystal structure analysis [34,35], cocrystal former screening [36,37], and cocrystal formation prediction [38,39]. For example, Mswahili et al. [40] extracted descriptors from SMILES and built several machine learning-based models for cocrystal prediction. The result showed that the artificial neural network outperformed other models. Our group also developed cocrystal prediction models based only on 2D structures obtained from CSD, which achieved high prediction performance and was also verified by a cocrystal screening experiment [41]. It cannot be ignored that traditional machine learning models can usually only handle fixed-size inputs, and feature extraction relies on feature engineering, which will lead to information loss problems [42]. Over the past few years, graph convolution networks (GCN) have become a practical and powerful tool for non-Euclidean structured data [43]. A molecule can naturally be considered a graph, whose atoms and bonds can be represented by nodes and edges. Therefore, it is more suitable to apply graphs to molecular representation and cocrystal prediction. Devogelaer et al. reported an ensemble approach combining a molecular fingerprint-based model and a molecular graph-based model for the prediction of cocrystal formation [44]. Recently, a powerful graph neural network (GNN) framework named CCGNet was developed for quickly predicting coformers in diverse cocrystal materials [45]. The results showed that CCGNet exhibited superior performance against seven competitive models and three challenging independent test sets, and one new energetic cocrystal predicted by CCGNet was successfully synthesized.

In previous studies, the datasets used to build the cocrystal prediction model were unbalanced (fewer negative samples), and the negative samples in the balanced dataset were usually from artificially generated data, which would result in false negative samples. Additionally, most models pay less attention to the noncovalent interactions between API and coformers. The aim of this study was first to collect a balanced cocrystal dataset in which negative samples included experimental sources and artificially generated samples, and then to develop a graph convolution network-based deep learning model, CocrystalGCN, for cocrystal prediction. After that, the CocrystalGCN was used to conduct virtual cocrystal screening of BEX from 109 coformers. We selected 30 top-scored coformers for experimental validation. The results showed that 4 out of 30 coformers successfully formed cocrystals with BEX. The physicochemical properties of these cocrystals were characterized using powder X-ray diffraction (PXRD), differential scanning calorimetry (DSC), thermogravimetric analysis (TGA), and so on. Finally, the pharmacokinetic study in rats was conducted to evaluate the in vivo absorption of BEX cocrystals and the free drug.

## 2. Materials and Methods

### 2.1. Data Collection and Processing

Datasets from various sources have been used to develop models in previous studies, most of which are limited data collected from the literature. The Cambridge Structural Database (CSD), a comprehensive database of organic and metal-organic molecule crystal structures, contains published cocrystal structures around the world [19]. Our group previously collected two-component crystal structures from CSD as positive samples to develop machine-learning classification models [41]. Here, the new version of CSD (version 2022.1.0., Cambridge Crystallographic Data Centre, Cambridge, UK) is filtered based on the same rules as reference [41] to obtain cocrystal data. Finally, 8016 cocrystal structures were obtained as positive samples (denoted a csd_pos). The same method as in reference [41], where different molecules are randomly combined into molecular pairs, was employed to generate 8016 negative samples (denoted as gen_neg). It is worth noting that false negative samples may be included in artificially generated negative samples. Recently, Jiang and partners collected 1052 negative samples generated by experiments from 186 literatures to minimize false negative samples [45]. Therefore, we obtained 1012 negative samples after removing duplicates, and then replaced the same number of artificially generated negative samples with the negative samples from experimental source (denoted as exp_neg) according to specific rules, to further minimize the false negative and build high-quality balanced dataset. A small subset was deleted from gen_neg in terms of the following steps:

1. Count the frequency of each compound in csd_pos and gen_neg;
2. Calculate the difference between the frequencies of each compound in gen_neg and csd_pos, rank them from large to small;
3. If the frequencies of top 1 compound in gen_neg and csd_pos are M and N, respectively, and M > N, use the MaxMin method [46] to select N molecule pairs from the M molecule pairs of top 1 compound in gen_neg, and delete the remaining molecules;
4. Repeat step 3 to top 2, top 3, …, top n compounds until 1012 artificially generated negative samples are removed, and then add the exp_neg.

**Figure 1:**
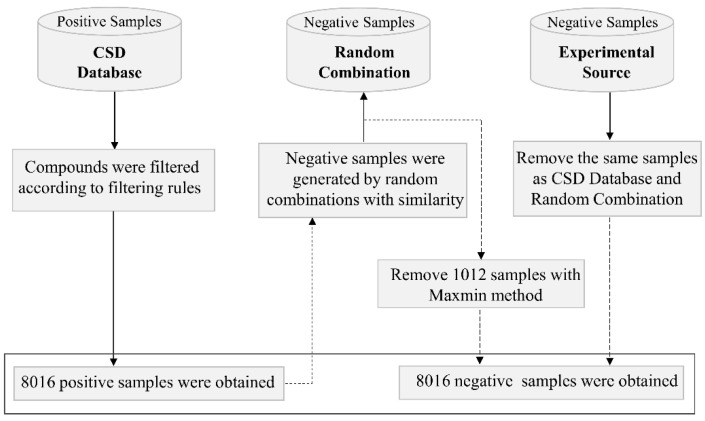
The whole workflow for dataset processing.

8016 negative samples including artificial or experimental samples were obtained. The whole diagram of dataset processing is shown in Figure 1.

### 2.2. Cocrystal Representation

For pharmaceutical cocrystals, the API interacts with coformer in a certain stoichiometric ratio by hydrogen bonds, π-π stacking, van der Waals, or other noncovalent bonds, rather than covalent bonds. Inspired by the work of Cho et al. [47], which utilizes two graphs to represent protein-ligand complex and are encoded as covalent and noncovalent adjacency matrices, we also apply the matrix strategy to cocrystal representation. Two-component cocrystal is first converted into an unweighted molecular graph in which each vertex represents an atom and each edge represents a bond. The adjacency matrix A is an *m* × *m* matrix, where $A_{ij}$ is either 0 or 1. $A_{ij}$ = 1 if ith and jth vertices are connected, and 0 if not. A can be divided into four smaller adjacency matrices, which encode the connection information of API-API ($A_{\mathrm{API}:\mathrm{API}}$), API-CF ($A_{\mathrm{API}:\mathrm{CF}}$), CF-API ($A_{\mathrm{CF}:\mathrm{API}}$), and CF-CF ($A_{\mathrm{CF}:\mathrm{CF}}$) atoms, respectively.

(1) $$A=\left[\begin{array}{ccc} A_{11} & \cdots & A_{1m}\\ \vdots & \ddots & \vdots \\ A_{m1} & \cdots & A_{mm} \end{array}\right]\;\;=\;\;\left[\begin{array}{cc} A_{\mathrm{API}:\mathrm{API}} & A_{\mathrm{API}:\mathrm{CF}}\\ A_{\mathrm{CF}:\mathrm{API}} & A_{\mathrm{CF}:\mathrm{CF}} \end{array}\right]$$

*m*: total number of atoms in the cocrystal; API: active pharmaceutical ingredient; CF: cocrystal former (Coformer).

**Figure 2:**
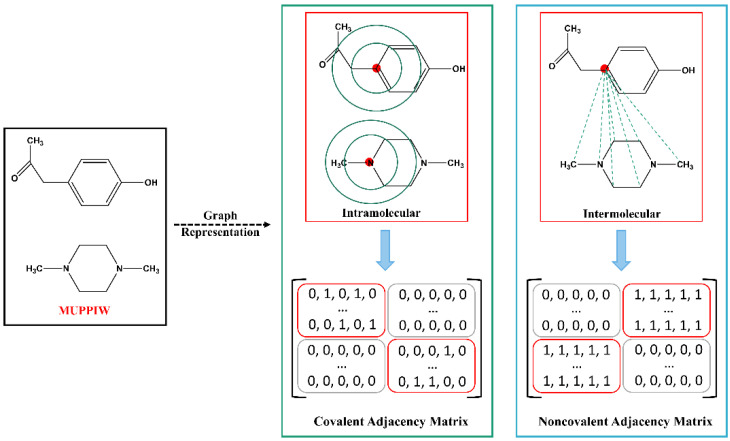
Schematic representation of covalent ($A_{C}$) and noncovalent ($A_{\mathrm{NC}}$) adjacency matrix.

Specifically, covalent and noncovalent adjacency matrix strategies were employed for cocrystal. As shown in Figure 2, the covalent and noncovalent adjacency matrices are in green and blue boxes, denoted as $A_{C}\;$ and $A_{\mathrm{NC}}$, respectively. $A_{C}$ represents the bond connectivity in API or coformer molecular graphs, in which $A_{\mathrm{API}:\mathrm{API}}$ and $A_{\mathrm{CF}:\mathrm{CF}}$ matrices are filled with 1 if there is a covalent bond between atoms, and 0 otherwise, $A_{\mathrm{API}:\mathrm{CF}}$ and $A_{\mathrm{CF}:\mathrm{API}}$ matrices are filled with 0. The $A_{\mathrm{NC}}$ represents all possible intermolecular noncovalent interactions between API and coformer, in which $A_{\mathrm{API}:\mathrm{CF}}$ and $A_{\mathrm{CF}:\mathrm{API}}$ matrices are filled with 1, and $A_{\mathrm{API}:\mathrm{API}}$ and $A_{\mathrm{CF}:\mathrm{CF}}$ matrices are filled with 0.

### 2.3. Architecture of the CocrystalGCN

Graph can be represented as G=(V, E, A), where V is a set of nodes, E is a set of edges, and A is a weighted or unweighted adjacency matrix. For *m*-nodes graph, the adjacency matrix, $\;A\in \;{\mathbb{R}}^{m\times m}$, denotes the following:

(2) $$A_{ij}=\;\{\begin{array}{c} a_{ij},\;\;\;\;if\;e_{ij}\in \;E\;\\ \;0,\;\;\;\;\;\;\;otherwise\; \end{array}$$

where $a_{ij}=1$ if the A is unweighted adjacency matrix. GNN has shown powerful performance in many drug discovery tasks [48]. GCN is one of GNN architecture variants, which apply the convolution operator to non-Euclidean structured data, such as molecule, social networks, and also have achieved state-of-the-art performance in classification and regression tasks [42].

**Figure 3:**
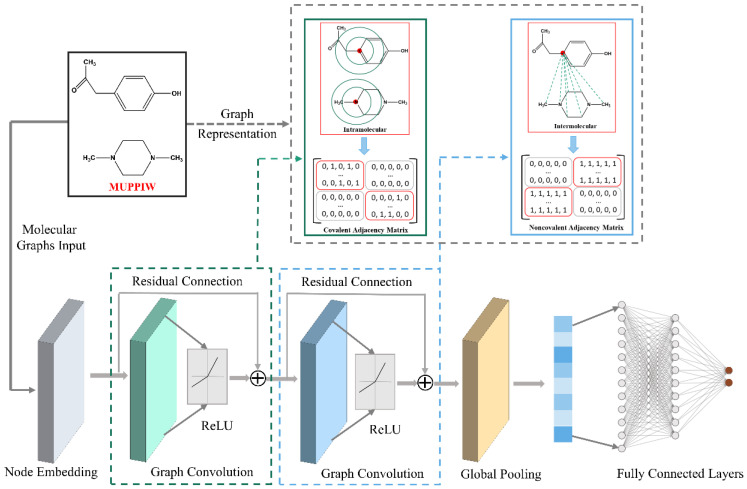
Illustration of CocrystalGCN architecture.

To better predict whether API and coformer can form cocrystal, a classification model named CocrystalGCN was developed. Similar to InteractionNet, CocrystalGCN contains five modules, among which two graph convolution modules adopt covalent ($A_{C}$) and noncovalent ($A_{\mathrm{NC}}$) adjacency matrix strategies mentioned above respectively. The whole framework of CocrystalGCN is shown in Figure 3. From the Figure 3, the five modules of CocrystalGCN are node embedding layers, graph convolutional layers with $A_{C}$ (GC_Ac_), graph convolutional layers with $A_{\mathrm{NC}}$ (GC_Anc_), graph pooling layer (GP), and fully connected layers (FC). In addition, there are two residual connection operations between graph convolutional layers. 

Let $X^{in}$ denote atomic feature matrix (detailed atom features are shown in Table S1), after the cocrystal molecular graphs are input into CocrystalGCN, the $X^{in}$ is first updated as node embedding $h^{NE}$ through node embedding layers,

(3) $$h^{NE}=\sigma \left(b_{2}+\sigma \left(b_{1}+X^{in}W_{1}\right)W_{2}\right)$$

Here and elsewhere in this paper, σ represents the activation function, is the Rectified Linear Units (ReLU). $b_{x}$ and $W_{x}$ are bias and the trainable parameter matrix, respectively. Once the node embedding layers propagation is finished, GC_Ac_ and GC_Anc_ layers receive node embedding $h^{NE}$ for local information aggregation. For each convolution step, the residual connection operations are used to updates representation additively. The detailed mathematics formulas of graph convolution are as follows:

(4) $$h_{c}=h^{NE}+\sigma \left(A_{C}h^{NE}W_{C}\right)$$

(5) $$h_{NC}=h_{c}+\sigma \left(A_{NC}h_{c}W_{NC}\right)$$

Cocrystal prediction belongs to graph-level classification task, so node-level atomic feature embedding need to be aggregated and generate a molecular vector in a permutation-invariant way. Therefore, $h_{NC}$ is aggregated into molecular vector $h_{GP}$,

(6) $$h_{GP}=1^{T}h_{NC}$$

After global pooling step, the molecular vector $h_{GP}$ is fed into fully connection layers and eventually outputs the probability of cocrystal formation between API and coformer.

(7) $$h_{0}=f\left(\sigma \left(b_{FC2}+\sigma \left(b_{FC1}+h_{GP}W_{FC1}\right)W_{FC2}\right)\right)$$

The 1 in Equation (6) stands for all-ones vector, function f() in Equation (7) is sigmoid activation function.

### 2.4. Performance Evaluation

To evaluate the effectiveness of various models, three predictive performance metrics are calculated between observed and predicted category labels according to true positive (TP), false positive (FP), true negative (TN), and false negative (FN) as follows: Precision, Recall, and Accuracy (Acc). The detailed calculation formulas are as follows:

(8) $$\mathrm{Recall}=\frac{\mathrm{TP}}{\mathrm{TP}+\mathrm{FN}}$$

(9) $$\mathrm{Peicision}=\frac{\mathrm{TP}}{\mathrm{TP}+\mathrm{FP}}$$

(10) $$\mathrm{Acc}=\frac{\mathrm{TP}+\mathrm{TN}}{\mathrm{TP}+\mathrm{TN}+\mathrm{FP}+\mathrm{FN}}$$

Another metric, the area under the receiver operating characteristic curve (AUC), is also employed to measure the binary classification capability. AUC value represents the performance of the model, the closer AUC value is to 1, the better the classification performance of the model, when AUC equals 0.5, the classification model is equivalent to a random classifier.

### 2.5. Model Training and Interpretation

The model attempts to differentiate positive coformers from whole coformer candidates, which can be regarded as a binary classification problem. Therefore, we adopted the binary cross-entropy shown below as loss function of the model, as follows:

(11) $$\begin{array}{c} Loss=-\frac{1}{N}\;\overset{N}{\underset{i=1}{\sum }}\left[y_{i}\cdot \log{\hat{y}}_{i}{}_{\;}+\left(1-y_{i}\right)\cdot \log\left(1-{\hat{y}}_{i}\right)\right] \end{array}$$

where N is the total number of samples, $y_{i}$ represents true label, and ${\hat{y}}_{i}$ represents the predicted probability of the *i*th sample. Adam [49] optimizer was used to minimize the loss function. Here, we implemented 10-fold cross-validation to evaluate prediction performance of the model. Dataset was randomly split into training set, validation set, and test set at the radio of 8:1:1. Hyperparameters searching was performed on the validation set. Table S2 shows the predefined hyperparameters search range. CocrystalGCN were implemented by using TensorFlow (version 2.8.0, Google, Mountain View, CA, America) [50]. 

Understanding why a model makes a certain decision can provide researchers with new and useful knowledge and make the model more acceptable [51,52]. In this work, we used a method named layer-wise relevance propagation (LRP) based decomposition [47,53] to explore the interpretation of CocrystalGCN model. The LRP method assumes that classifier can be decomposed into several layers, and the relevance of each neuron is calculated through reversely propagating of network (from output to input layer). The contribution of a neuron to the prediction result can be represented by relevance. Here, we can obtain the contribution of each atom in the compound to the cocrystal score predicted by CocrystalGCN through LRP analysis and generate the heat map of atomic contribution. By analyzing the heat map and the corresponding experimental results, we can gain a deeper understanding of the basis for the model predictions.

### 2.6. Prediction of Cocrystal of BEX

Due to the large number of coformers, the cocrystal screening experimental methods are expensive and time-consuming. In the present study, we applied the established CocrystalGCN model to predict coformer of BEX.

In total, we collected 109 coformer candidates based on the generally recognized as safe (GRAS) list and in-house coformer database. The compound pairs consisting of BEX and coformer candidates were fed into CocrystalGCN model. The cocrystal probability generated by CocrystalGCN model was ranked from large to small, and the top 30 coformers were selected for cocrystal screening experiment.

### 2.7. Materials

The commercially available BEX was purchased from Shanghai Titan Scientific Co., Ltd. (Shanghai, China). The analytical grade of solvents used for crystallization experiments was obtained from Sinopharm Chemical Reagent Co., Ltd. (Shanghai, China) and used as received.

### 2.8. Preparation of BEX Cocrystals

The cocrystals of BEX were synthesized by the method of liquid-assisted grinding (LAG). BEX (0.1 mmol) and stoichiometric amounts of the four corresponding coformers were ground for about 20 min at 30 Hz after adding 10 μL of methanol. A Retsch MM200 device was utilized to conduct the grinding experiments. Then, the resulting samples were detected by PXRD to confirm the formation of cocrystals.

### 2.9. Preparation of Single Crystals

The saturated solution of API was obtained by adding the excess BEX to the 2 mL of ethanol solvent and it was stirred for 12 h. Then the BEX-saturated solution was filtered and the excess coformers were added to the solution to stir overnight to produce the cocrystals. After that, the resulting samples were filtered and the single crystals were obtained by cooling the final filtrate at 4 °C for about a week.

### 2.10. Powder X-ray Diffraction (PXRD)

A Bruker D8 Advance X-ray diffractometer was utilized to obtain the PXRD patterns. The voltage was set to 40 kV and the current was set to 40 mA. Data were collected in the 3–40° 2θ range at a scan rate of 5°/min at ambient temperature. Data were imaged and integrated using RINT Rapid, and peaks were analyzed using Rigaku’s Jade 6.0. A corindon standard was used to calibrate the instrument.

### 2.11. Single Crystal X-ray Diffraction (SCXRD)

SCXRD experiments were performed on an APEX II CCD diffractometer (Bruker) with Mo Kα radiation (λ = 0.71073 Å). Mounting a suitable crystal on the loop and diffraction was performed at 170 K. The integration and scaling of the intensity data were operated on the SAINT program, and SADABS was used to correct the data for absorption effects. The direct methods on SHELXTL were used to solve and full matrix least squares were used to refine the crystal structure by OLEX2. The CCDC numbers are 2,203,843—2,203,846 for BEX-2,5-dimethylpyrazine, BEX-pyrazine, BEX-methyl isonicotinate, BEX-ethyl isonicotinate., respectively.

### 2.12. Thermal Analyses

A DSC TA Q2000 instrument was utilized to conduct the DSC experiments with a nitrogen flow purged at 50 mL min^−1^. Solid powder weighing 4−8 mg was heated in sealed aluminum pans, and the heating rate is 10 °C min^−1^. A Netzsch TG 209F3 apparatus was utilized to conduct the Thermogravimetric analysis, and the samples were heated at 10 °C min^−1^ to 400 °C. 

### 2.13. Fourier Transformation Infrared (FTIR) 

A Nicolet-Magna FT-IR 750 spectrometer was utilized to obtain the FTIR spectra in the range from 350 to 4000 cm^−1^, with a resolution of 4 cm^−1^ at ambient conditions.

### 2.14. High-Performance Liquid Chromatography 

The concentration of BEX samples was detected by the Agilent 1260 Series Infinite HPLC. The Eclipse XDB-C18 column (4.6 × 150 mm, 5 μm) was used to separate the analytes. The column temperature was set at 30 °C and the injection volume was 10 μL. The mobile phase was composed of 0.1% trifluoroacetic acid in water and acetonitrile (10:90, *v*/*v*) pumped at 1.0 mL/min. The 260 nm was set as the UV-vis detection wavelength.

### 2.15. Solubility and Powder Dissolution

Excess amounts (10 mg) of BEX and four cocrystals samples were added to the 4 mL of pH 2.0, 4.5, and 6.8 buffer solutions respectively, with the presence of 0.05% CTAB (hexadecyl trimethyl ammonium bromide). Then the saturated solutions were stirred at 300 rpm for one day at 37 °C. The equilibrium solubility of BEX was determined by HPLC after filtering the solutions.

The solid samples of BEX and four cocrystals were sieved through 100-mesh sieves. In total, 20 mg of BEX and corresponding cocrystal powders (n = 3) were added to pH 6.8 phosphate buffer with the presence of 0.05% CTAB at 37 °C, rotating at 100 rpm. The samples were collected at 5, 10, 15, 20, 30, 40, 50, and 60 min. The concentration was measured by HPLC.

### 2.16. Pharmacokinetics in Rats

The PK studies were performed according to the Guide for Care and Use of Laboratory Animals and were approved by the Institutional Animal Care and Use Committee of Shanghai Institute of Materia Medica. There were two groups of male Sprague−Dawley rats and the number of rats is six in each group. The 0.5% CMC-Na suspension formulations of BEX bulk powder, BEX-pyrazine, and BEX-2,5-dimethylpyrazine were prepared for the drug administration. The dose is 10 mg/kg BEX. The animals had free access to water and fasted overnight before dosing. After the administration, the 600 μL blood sample was collected at 0.5, 1, 2, 3, 4, 6, and 8 h. Plasma was separated by centrifugation (14,000 rpm, 10 min) and stored at −80 °C. In total, 200 μL of rat plasma sample was mixed with 600 μL acetonitrile through a vortex mixer for 20 min and centrifuged for 5 min (14,000 rpm). The BEX concentration was measured by the SCIEX Triple Quad™ 4500 LC-MS instrument. The analytes were separated by the Supelco Ascentis Express C18 column (2.1 × 50 mm, 2.7 μm). The column temperature was set at 35 °C. The mobile phase was composed of 0.2% formic acid in water (solvent A) and acetonitrile (solvent B) pumped at 0.6 mL/min. The gradient elution program started with 55% B and increased linearly to 90% B at 1.5 min and held for 0.5 min. It returned to the 55% B at the 3 min and held for 2 min. The injection volume was 10 μL, and the UV-vis detection wavelength was set at 260 nm. The negative ion detection mode was operated in the mass spectrometer. Multiple reaction monitoring modes (MRM) was used to perform the detection and quantification, with m/z 347.3→303.0 for BEX. The DAS 2.0 program was used to perform the PK analysis.

## 3. Results and Discussion

### 3.1. Dataset Analysis

**Figure 4:**
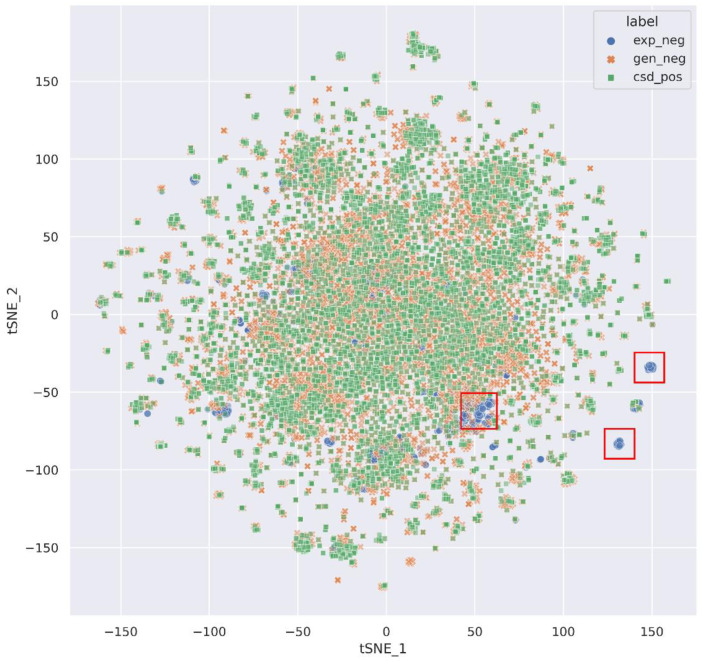
Chemical space distribution of exp_neg (blue), gen_neg (orange), and csd_pos (green) dataset by t-SNE method using ECFP fingerprint. The red box mainly highlights blue points that do not overlap with orange and green points.

To illustrate the advantages of the composition of the negative dataset, we visualized the chemical space distribution of exp_neg (blue), gen_neg (orange), and csd_pos (green) datasets based on 1024 bits extended-connectivity fingerprint (ECFP) [54] by the t-distribution stochastic neighbor embedding (t-SNE) method [55] in Figure 4. From the figure, it is observed that the chemical space distribution of compounds in gen_neg is similar to the csd_pos, which can easily be explained by the generation of gen_neg based on csd_pos. In contrast, the chemical space distribution of compounds in exp_neg is not completely contained in gen_neg and csd_pos, and the different points are mainly concentrated in the red box. This phenomenon shows that negative samples generated by experiments can extend the chemical diversity of artificially generated negative samples; additionally, it may also reduce the number of false negative samples. Therefore, the exp_neg and gen_neg were combined to form the final negative sample dataset. The ECFP fingerprint was calculated by the RDKit library [56] and t-SNE was implemented by Scikit-learn (version 1.0.2) [57]. 

### 3.2. Comparison of Prediction Performance between CocrystalGCN and Baselines

In order to compare the performance of CocrystalGCN, we also constructed several models based on both machine learning and deep learning methods. Three machine learning methods are the following: support vector machine (SVM) [58], random forest (RF) [59], and eXtreme gradient boosting (XGB) [60]. The latter two methods belong to the category of ensemble learning methods, which have been widely used in various tasks and achieved great results [61]. Deep neural networks (DNN) [62] and DeepDDS [63] are deep learning methods. A DNN typically includes an input layer, hidden layers, and an output layer, each of which consists of a different number of neurons. These classical machine learning-based models and DNN were implemented by using Scikit-learn (version 1.0.2), and predefined hyperparameters are shown in Table S3. The DeepDDS model was created for drug-drug synergy prediction, and its input is the embedding of two compounds and the gene expression profile of a cell line. Here, we removed the MLP module in the DeepDDS that encodes cell line features, leaving the rest unchanged. The difference between CocrystalGCN and revised DeepDDS is the way in which the representation of compounds is encoded. The embedding of two compounds in DeepDDS is encoded by two equivalent GCNs and then concatenated together, while in CocrystalGCN, the adjacency matrix of two compounds is concatenated together through different strategies and then input into a GCN layer with a feature matrix. Additionally, we also performed ablation experiments of CocrystalGCN by removing the GC_Ac_ (CocrystalGCN_C) and GC_Anc_ (CocrystalGCN_C) layers separately to observe their influences on model performance.

We conducted 10-fold cross-validation to evaluate the predictive performance of each model. The whole dataset was randomly divided into training, validation, and test sets in an 8:1:1 ratio in each cross-validation experiment. The average predictive performance metrics of 10-fold were obtained as a final result of the model. Table 1 shows the statistical results of CocrystalGCN with its variants and baselines on the validation set and test set. As shown in Table 1, CocrystalGCN achieved pretty great performance in the Acc and Precision metrics both on the validation set and test set. DeepDDS achieved the best AUC value (0.871) on the test set, while RF achieved the best AUC value (0.884) on the validation set. It is worth noting that other performance metrics of RF also achieved competitive results compared with other models, which indicates the powerful predictive ability of RF. A previous study [41] on cocrystal prediction also found that the prediction performance of RF was better than other classical machine learning methods. Overall, the predictive performance of CocrystalGCN generally outperforms baselines.

To further explore how different adjacency matrix strategies affect the predictive ability of CocrystalGCN, we conducted an ablation experiment. CocrystalGCN_C and CocrystalGCN_NC represent the GCN layer of CocrystalGCN, containing GCAc and GCAnc, respectively, while CocrystalGCN contains both. From Table 1, we can find that CocrystalGCN and CocrystalGCN_NC outperform CocrystalGCN_C both on the validation set and the test set, which indicates that graph convolution modules adopting a noncovalent adjacency matrix strategy (GCAnc layer) is more important than those with a covalent adjacency matrix strategy (GCAc layer) for model performance. In other words, the GCAnc layer can well simulate the interactions between API and coformer, and this noncovalent interaction is necessary for the cocrystal formation of API and coformer. In short, our CocrystalGCN model generally outperforms its variants. 

### 3.3. Interpretation of the CocrystalGCN

**Figure 5:**
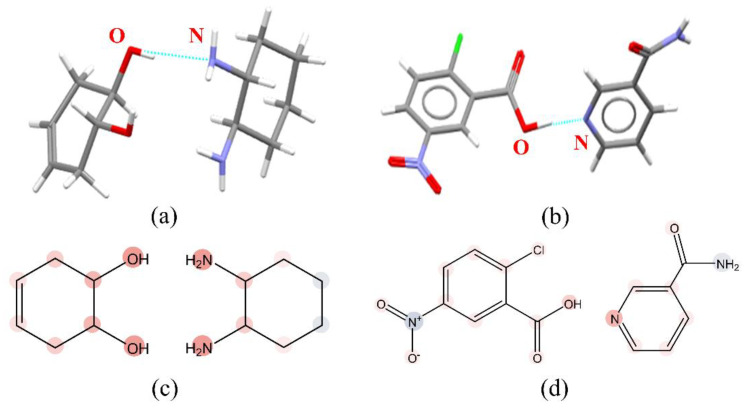
The 3D interaction modes and heat maps of two cocrystals: (**a**) Interaction mode diagram of cocrystal with CSD code GUCQUQ, (**b**) interaction mode diagram of cocrystal with CSD code NUBHAW. The gray, blue, red, green, and white represent carbon, nitrogen, oxygen, chlorine, and hydrogen atoms, respectively. The cyan lines represent hydrogen bonds, (**c**) Heat map for atomic contribution generated by LRP method, GUCQUQ, (**d**) Heat map for atomic contribution generated by LRP method, NUBHAW. The red, blue, and white represent positive influence, negative influence, and zero influence, respectively. Color intensity represent different contributions, the contribution increases with the darker color.

For exploring the interpretation of the CocrystalGCN, two cocrystals with CSD code GUCQUQ and NUBHAW from the test set were sampled, and conducted to LRP analysis. The intermolecular interaction mode in two cocrystals from the CSD database and the heat map obtained from trained CocrystalGCN are shown in Figure 5. For the cocrystal GUCQUQ, the three-dimensional intermolecular interaction mode (Figure 5a) shows a hydrogen bond formed between the hydroxyl group of 4-cyclohexene-1,2-diol and the amino group of 1,2-Cyclohexanediamine. The corresponding heat map of atomic contribution (Figure 5c) shows that the oxygen atom of the hydroxyl group and nitrogen atom of the amino group of the above two compounds are assigned the deepest red colors, respectively, indicating that the two atoms have the largest positive contribution to the predicted score, which is consistent with the experimental result. A similar result was obtained for another cocrystal NUBHAW. The interaction mode (Figure 5b) shows a hydrogen bond formed between the hydroxyl group contained in the carboxyl group of 2-chloro-5-nitrobenzoic acid and nitrogen on pyridine in nicotinamide, and the corresponding heat map of atomic contribution (Figure 5d) shows the hydroxyl oxygen on the carboxyl group and the nitrogen atom on the pyridine in a deeper red color. The other nitrogen atom in nicotinamide is assigned a blue color and generates a negative influence on prediction. The high correlation between LRP analysis and knowledge-based results indicates that our CocrystalGCN model can learn some knowledge related to cocrystal formation, and the LRP method applied in CocrystalGCN can help us understand why the model makes a decision. 

### 3.4. Results of Virtual Screening Based on CocrystalGCN

**Figure 6:**
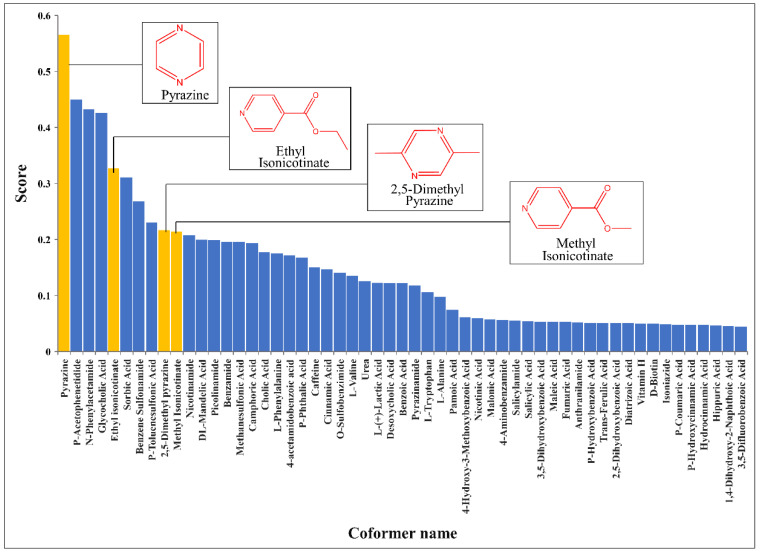
Score ranking of top 55 coformers forming cocrystals with bexarotene by the CocrystalGCN. Four experimentally verified coformers capable of forming cocrystals with BEX are marked in yellow, which are pyrazine, ethyl isonicotinate, 2,5-dimethyl pyrazine, and methyl isonicotinate, respectively.

The solubility of BEX is poor. We expect to improve its solubility with the cocrystal method. The optimal CocrystalGCN model trained on the whole dataset was used to predict the cocrystal formation of BEX. The score of cocrystal formation with BEX of 109 coformer candidates was predicted. The corresponding scores of the top 55 coformer candidates are displayed as a bar chart in Figure 6 because of space limitations. From the figure, the top 10 coformers and corresponding scores are pyrazine (0.565), p-acetophenetidide (0.450), n-phenylacetamide (0.432), glycocholic acid (0.426), ethyl isonicotinate (0.327), sorbic acid (0.311), benzene sulfonamide (0.269), p-toluenesulfonic acid (0.230), 2,5-dimethyl pyrazine (0.217), and methyl isonicotinate (0.214). 

The prediction results show that the coformers after the top 30 all have scores below 0.1. Therefore, in order to quickly discover new cocrystals of BEX, the top 30 coformers were selected to perform cocrystal experimental screening through the LAG method. From the results of LAG, four coformers formed cocrystals with BEX, which are pyrazine, ethyl isonicotinate, 2,5-dimethyl pyrazine, and methyl isonicotinate. Other coformers did not form cocrystals with BEX, and the resulting samples were the physical mixtures of BEX and coformers. The chemical structures of four coformers with their score bars in yellow are displayed in Figure 6. The four coformers are ranked first, fifth, ninth, and tenth in the scoring list, respectively, demonstrating that the prediction performance of CocrystalGCN is highly accurate. Particularly, we also observed that pyrazine and 2,5-dimethyl pyrazine, ethyl isonicotinate, and methyl isonicotinate are two groups of homologues, and their structures are highly similar. This phenomenon of similarity can be explained by the fact that our model, in addition to learning the natural crystallization mechanism, also takes into account the contribution of structural similarity of coformers to cocrystal formation. Springuel et al. [64] pointed out that molecules having relatively similar chemical structures are more likely to form cocrystals with the same coformer in their study.

### 3.5. PXRD and Thermal Analysis of Cocrystals

All of the samples obtained by grinding were detected by PXRD to determine the BEX new phases (Figure S1). The patterns of the resulting samples are significantly different from those of BEX raw materials or coformers, confirming the formation of cocrystal phases. Take BEX-pyrazine as an example. The characteristic peaks of BEX at 2θ = 4.81°, 11.51°, 12.55°, 14.58°, 14.81°, 18.82°, 23.22°, 24.46°, and the characteristic peaks of pyrazine at 2θ = 17.53°, 18.32°, 19.03°, 25.23°, 28.30°, and 31.30° disappeared, while the characteristic peaks of cocrystal appeared at 2θ = 5.76°, 11.54°, 13.78°, 14.72°, 15.82°, 16.08°, 17.52°. The measured peaks of all cocrystals are in good agreement with the simulated patterns, indicating the formation of high-phase purity cocrystals.

**Figure 7:**
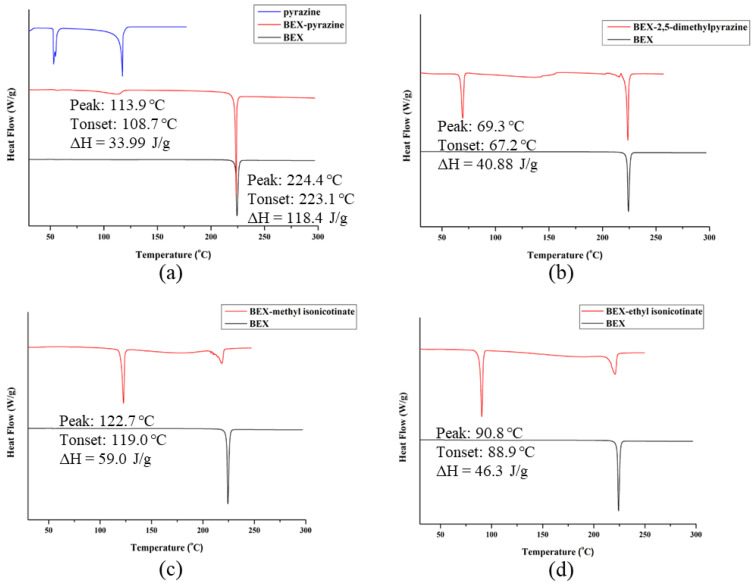
DSC profiles of solid forms of BEX: (**a**) BEX-pyrazine, (**b**) BEX-2,5-dimethylpyrazine, (**c**) BEX-methyl isonicotinate, and (**d**) BEX-ethyl isonicotinate.

**Figure 8:**
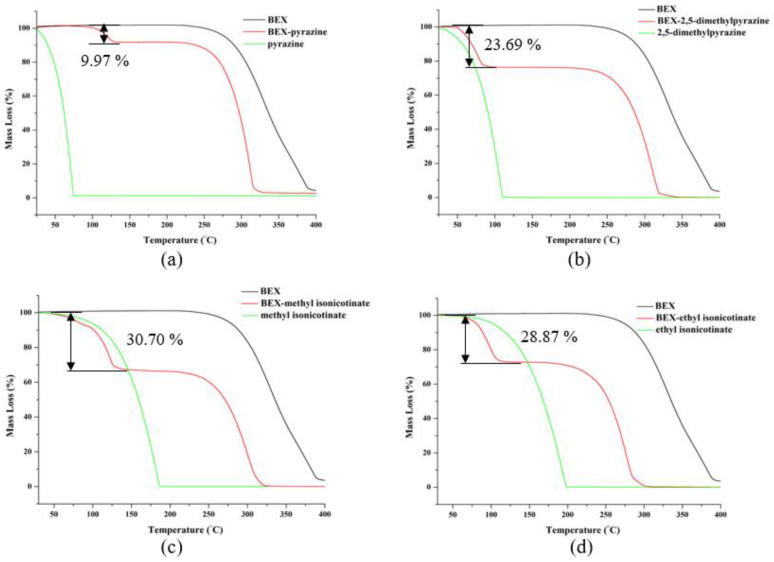
TG patterns of solid forms of BEX: (**a**) BEX-pyrazine, (**b**) BEX-2,5-dimethylpyrazine, (**c**) BEX-methyl isonicotinate, and (**d**) BEX-ethyl isonicotinate.

The DSC curves (Figure 7) show that the commercially available BEX powder possesses a high crystallinity with a melting point of 224.4 °C. For the cocrystal of BEX-pyrazine, it represents a small endothermic peak at about 113.9 °C, corresponding to the release of pyrazine from the crystal structures during the heating process. This is also evidenced by the TG plot in Figure 8a, where a weight loss of 9.97%, equivalent to half a pyrazine molecule, is observed. Once the coformer is released from the cocrystal structure, the remaining solid is the starting material BEX, accounting for the appearance of the endothermic peak at 224.4 °C on the DSC curve of BEX-pyrazine. The cocrystal of BEX-2,5-dimethylpyrazine, it experienced the same thermal event as that of BEX-pyrazine and showed a mass loss of 23.69% in TG (Figure 8b), indicating a 1:1 molar ratio of BEX to 2,5-dimethylpyrazine. For the cocrystals of BEX-methyl isonicotinate and BEX-ethyl isonicotinate, the coformers are lost at 122.7 °C and 90.8 °C, respectively. Additionally, according to the TG results, the stoichiometric ratio of API and conformer in these two cocrystals is 1:1.

### 3.6. Crystal Structure Analysis

**Figure 9:**
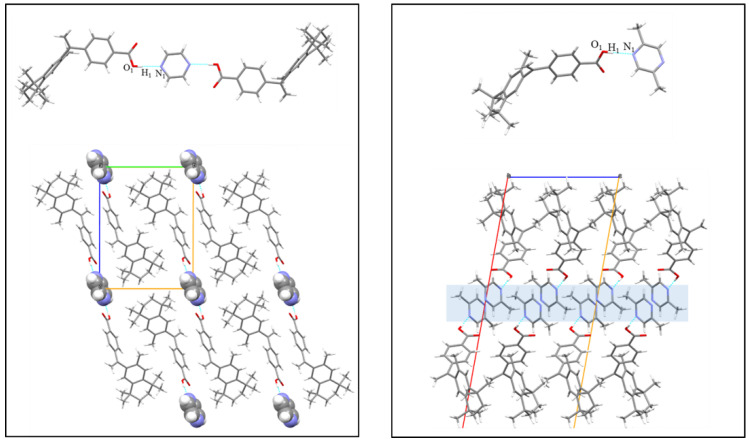
Packing modes of BEX-pyrazine (**left**) and BEX-2,5-dimethylpyrazine (**right**).

Crystallographic data of cocrystals are listed in Table S4. BEX-pyrazine (Figure 9) was solved in the triclinic P-1 space group with one BEX molecule and half a molecule of pyrazine in the asymmetric unit. BEX connects with pyrazine molecules (O1−H1⋯N1, 2.723 Å) to form the centrosymmetric structure. Two adjacent BEX molecules are separated by another antiparallel BEX molecule to form the 2D buckle structure. The 2D layers are alternatively packed along the *a*-axis to form the 3D structure via interlayer π-π interactions.

The cocrystal of BEX-2,5-dimethylpyrazine belongs to the monoclinic C 2/c space group, and the asymmetric unit contains a molecule of BEX and a molecule of 2,5-dimethylpyrazine. The carboxyl groups in BEX molecules form an intermolecular hydrogen bond with 2,5-dimethylpyrazine molecules via O1−H1···N1 (d = 2.726 Å). In the *ac* plane, the hydrophilic part is composed of 2,5-dimethylpyrazine molecules stacked along the c-axis. Additionally, BEX molecules are interspersed on both sides of the hydrophilic part to form the 2D structure. Through the π-π interactions, the 2D structures extend along the *b*-axis to form 3D structures. The cocrystals of BEX-methyl isonicotinate and BEX-ethyl isonicotinate stack in a similar manner as that of BEX-2,5-dimethylpyrazine.

### 3.7. Solubility and Powder Dissolution

**Figure 10:**
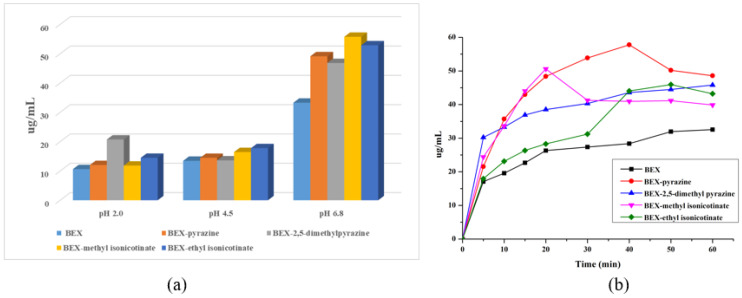
(**a**) Equilibrium solubility of BEX and cocrystals. (**b**) Powder dissolution of BEX and cocrystals at pH 6.8. The 0.05% CTAB was added in the buffer solution.

The solubility is one of the most important physicochemical properties for a drug to achieve a desired concentration in systemic circulation [65]. As a BCS Class II compound, the absorption of BEX is limited by its low solubility. Additionally, the equilibrium solubility of BEX and four cocrystals was measured in the presence of 0.05% CTAB in buffer solutions at pH 2.0, 4.5, and 6.8, as shown in Figure 10a. The solubility of BEX was less than 10 ug/mL at pH 2.0, indicating it was almost insoluble in the stomach. However, the cocrystal BEX-2,5-dimethylpyrazine presented a 2-fold superiority (20 ug/mL at pH 2.0) over the parent BEX bulk powder. Being a weakly acid drug, the solubility of BEX increased with the increasing pH. Therefore, the solubility of BEX was slightly higher than 10 ug/mL at pH 4.5 and it increased to about 30 ug/mL at pH 6.8. For the four cocrystals, the solubility did not show a significant difference with BEX raw materials at pH 4.5. However, all cocrystals presented about a 1.5-fold advantage compared to the parent BEX at pH 6.8. The PXRD patterns of residues after equilibrium solubility studies were shown in Figure S3, and the pH values of the final solutions were shown in Table S5. The results indicated that all cocrystals dissociated and the remaining solid was the raw material BEX. The pH values of the solutions after the solubility experiments did not change much.

The powder dissolution profiles (Figure 10b) indicated that BEX exhibited poor dissolution performance, with a measured solubility of about 30 ug/mL after 60 min at pH 6.8. All cocrystals presented higher solubility than BEX in all periods. The spring-parachute effect was observed in the dissolution of BEX-pyrazine and BEX-methyl isonicotinate, which showed the highest solubility at 40 and 20 min, respectively. The maximum solubility values of BEX-pyrazine, BEX-2,5-dimethylpyrazine, BEX-methyl isonicotinate, and BEX-ethyl isonicotinate were 1.8, 1.4, 1.6, and 1.4 times higher than those of BEX, respectively. The dissolution advantage of cocrystals over the parent BEX under the condition of pH 6.8 indicated that the absorption of BEX in the posterior part of the small intestine could be improved through the cocrystal formation. The PXRD patterns of residues after powder dissolution were shown in Figure S4 and the pH values of the final solutions were shown in Table S6. The conditions of the cocrystal dissociation were the same as those of the solubility experiments.

### 3.8. PK Studies in Rats

**Figure 11:**
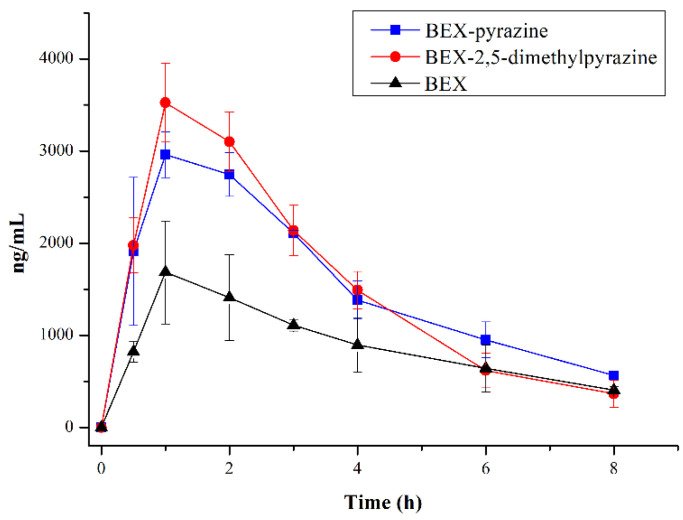
The PK profiles of BEX and cocrystals in rats.

Owing to the improved solubility and dissolution of BEX by forming cocrystals, we hypothesize that the in vivo absorption can also be enhanced. Additionally, the edible cocrystals of BEX-pyrazine and BEX-2,5-dimethylpyrazine were chosen to perform the pharmacokinetic study in rats. The plasma concentration−time profiles of BEX and cocrystals are presented in Figure 11, and the mean pharmacokinetic parameters are listed in Table 2. The results reveal that the C_max_, AUC_0−8h_, and AUC_inf_ of cocrystals are statistically different from those of free BEX. The C_max_ of BEX-pyrazine and BEX-2,5-dimethylpyrazine are 1.8 and 2.1 times, respectively, as that of pure BEX, and the plasma exposures (AUC_0−8h_) of BEX-pyrazine and BEX-2,5-dimethylpyrazine show a 1.7 and 1.8-fold improvement compared to the commercially available BEX powder. The PK studies demonstrate that the oral absorption of BEX can be indeed enhanced, originating from the improved solubility and dissolution of these two cocrystals.

## 4. Conclusions

In summary, a balanced dataset containing 8016 positive and 8016 negative samples was collected. CocrystalGCN was trained for cocrystal prediction based on the dataset. The AUC, Acc, Precision, and Recall values of the CocrystalGCN classification model were 0.866, 0.811, 0.802, and 0.830, respectively. After that, we utilized the optimum CocrystalGCN model for virtually screening the cocrystals of BEX. Based on the combination of virtual and experimental screening of BEX, we successfully obtained four new cocrystals of BEX. The PXRD patterns and the thermal behaviors of different solid forms were investigated. The intermolecular interactions in the single-crystal structures were clarified. All cocrystals presented superior solubility and dissolution over the parent BEX in the buffer solutions. Additionally, the PK studies indicate that the bioavailability of BEX-pyrazine and BEX-2,5-dimethylpyrazine is 1.7 and 1.8 times, respectively, that of the commercially available BEX powder. It indicates that our predictive model is effective to guide the cocrystal design and the absorption issues of water-insoluble drugs can be addressed through the cocrystal strategy.

## Figures and Tables

**Table 1:** Performance comparison of CocrystalGCN and baselines on validation and test set.

Models	Validation Set				Test Set			
	AUC	Acc	Precision	Recall	AUC	Acc	Precision	Recall
RF	**0.884**	0.801	0.797	0.808	0.795	0.795	0.793	0.801
SVM	0.852	0.787	0.770	0.817	0.783	0.783	0.767	0.818
XGBoost	0.855	0.781	0.770	0.802	0.780	0.780	0.775	0.792
DNN	0.839	0.766	0.757	0.782	0.759	0.760	0.739	0.806
DeepDDS	0.879	0.810	0.789	0.844	**0.871**	0.805	0.787	0.838
CocrystalGCN_C ^1^	0.855	0.803	0.782	0.829	0.854	0.794	0.784	0.812
CocrystalGCN_NC ^2^	0.853	0.816	0.795	**0.854**	0.855	0.806	0.785	**0.840**
CocrystalGCN ^3^	0.866	**0.818**	**0.802**	0.845	0.866	**0.811**	**0.802**	0.830

^1^ Only contains GCAc layer in CocrystalGCN; ^2^ Only contains GCAnc layer in CocrystalGCN; ^3^ Containing both GCAc and GCAnc layers in CocrystalGCN. The best performance is in bold.

**Table 2:** Pharmacokinetic parameters of BEX and cocrystals in rats.

Parameters	BEX	BEX-Pyrazine	BEX-2,5- Dimethylpyrazine
T_1/2_ (h)	2.98 ± 1.18	2.59 ± 0.10	1.74 ± 0.39
C_max_ (μg/L)	1682.67 ± 559.27	2960.33 ± 248.02 *	3577.00 ± 387.34 *
AUC_0−8h_ (h·μg/L)	7215.17 ± 810.61	12561.45 ± 919.13 *	12702.38 ± 978.30 *
AUC_inf_ (h·μg/L)	8792.75 ± 1076.36	14656.33 ± 1085.66 *	13513.29 ± 1358.85 *

* *p* < 0.05 compared with BEX. Data are means ± SD.

## Data Availability

The results obtained for all experiments performed are shown in the manuscript and Supplementary Materials, the raw data will be provided upon request.

## Supplementary Materials

The following supporting information can be downloaded at: https://www.mdpi.com/article/10.3390/pharmaceutics14102198/s1, Figure S1: PXRD patterns of solid forms of BEX: (a) BEX-pyrazine, (b) BEX-2,5-dimethylpyrazine, (c) BEX-methyl isonicotinate, and (d) BEX-ethyl isonicotinatetitle; Figure S2: FTIR spectra of (a) BEX-pyrazine, (b) BEX-2,5-dimethylpyrazine, (c) BEX- methyl isonicotinate, and (d) BEX-ethyl isonicotinate; Figure S3: XRPD patterns of residues after equilibrium solubility studies: (a) pH 2.0, (b) pH 4.5, and (c) pH 6.8. The XRPD pattern from top to bottom in each figure is BEX-pyrazine, BEX-2,5-dimethylpyrazine, BEX-methyl isonicotinate, BEX- ethyl isonicotinate, and BEX raw materials, respectively; Figure S4: XRPD patterns of residues after powder dissolution: The XRPD pattern from top to bottom is BEX-pyrazine, BEX-2,5-dimethylpyrazine, BEX-methyl isonicotinate, BEX-ethyl isonicotinate, and BEX raw materials, respectively; Table S1: Atom features used in CocrystalGCN; Table S2: The search spaces of hyperparameters for CocrystalGCN; Table S3: The search spaces of hyperparameters for baseline models; Table S4: Crystallographic Data of BEX cocrystals; Table S5: The pH values of Bex and cocrystal solutions after equilibrium solubility experiments (n = 3); Table S6: The pH values of Bex and cocrystal solutions after powder dissolution at pH 6.8 (n = 3).
