# Supplementary material for: Cocrystal Prediction of Bexarotene by Graph Convolution Network and Bioavailability Improvement
Source: Pharmaceutics. 2022 Oct 16;14(10):2198. doi: 10.3390/pharmaceutics14102198 (PMC9611166; doi:10.3390/pharmaceutics14102198)
Supplement: Supplementary file 1 [file pharmaceutics-14-02198-s001.zip › pharmaceutics-1956877-supplementary.pdf]

Cocrystal Prediction of Bexarotene by Graph Convolution Network and Bioavailability Improvement

Supporting Information

Fu Xiao [1,2,†], Yinxiang Cheng [1,3,†], Jian-Rong Wang [1], Dingyan Wang [1,3], Yuanyuan Zhang [1,3], Kaixian Chen [1,2,3],
Xuefeng Mei [1,3,*] and Xiaomin Luo [1,2,3,*]

[1] State Key Laboratory of Drug Research and Drug Discovery and Design Center, Pharmaceutical Analytical & Solid-State Chemistry Research Center, Shanghai Institute of Materia Medica,
Chinese Academy of Sciences, Shanghai 201203, China
[2] School of Chinese Materia Medica, Nanjing University of Chinese Medicine, Nanjing 210023, China
[3] University of Chinese Academy of Sciences, Beijing 100049, China
* Correspondence: xuefengmei@simm.ac.cn (X.M.); xmluo@simm.ac.cn (X.L.)
† These authors contributed equally to this work.

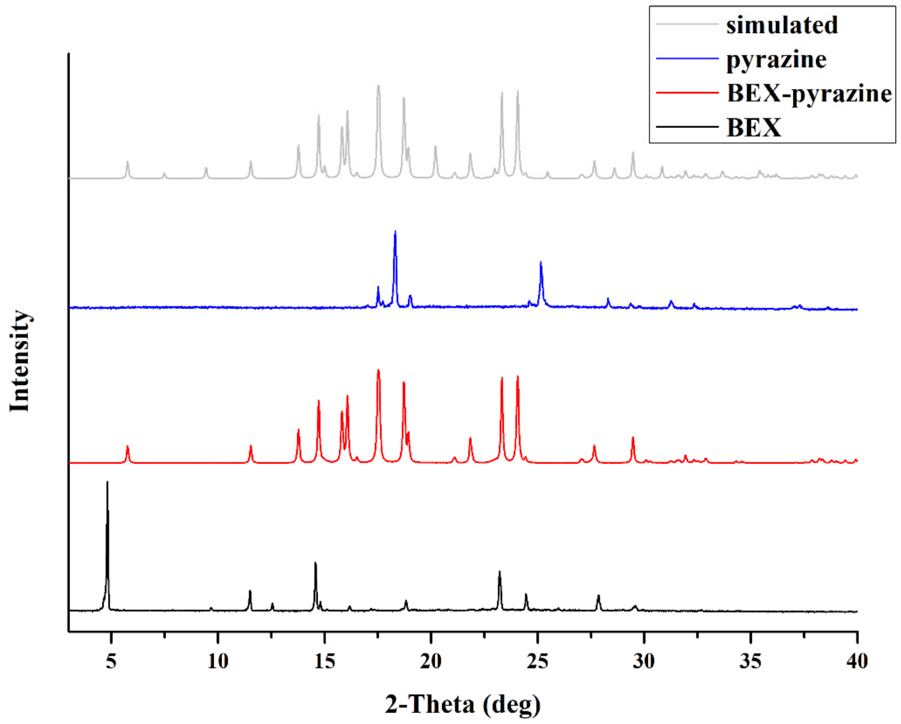
simulated
pyrazine
BEX-pyrazine
BEX
Intensity
5
10
15
20
25
30
35
40
2-Theta (deg)

（a）

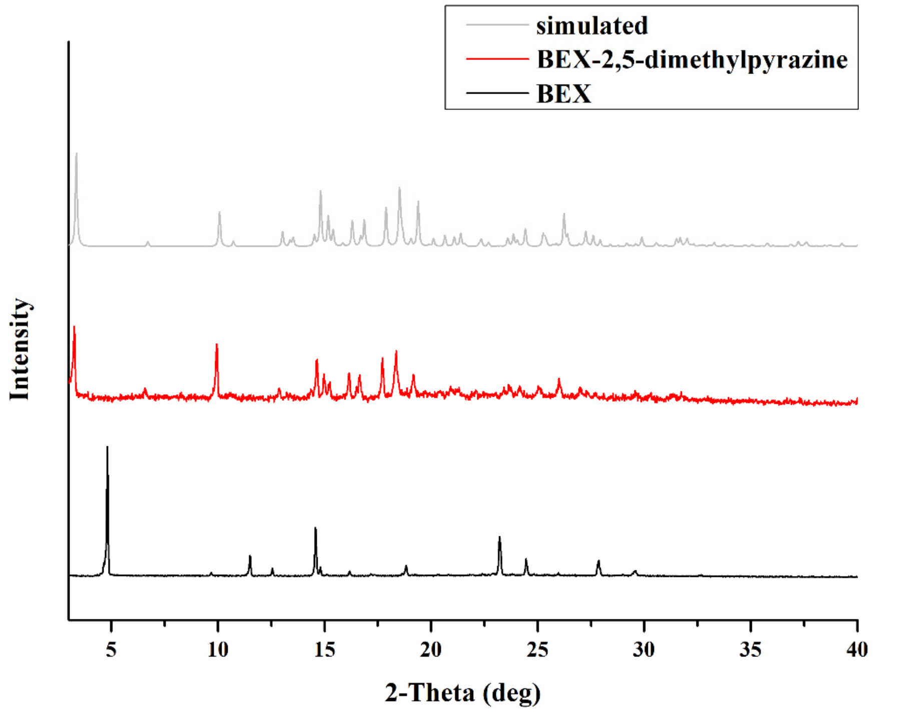
simulated
BEX-2,5-dimethylpyrazine
BEX
Intensity
5
10
15
20
25
30
35
40
2-Theta (deg)

（b）

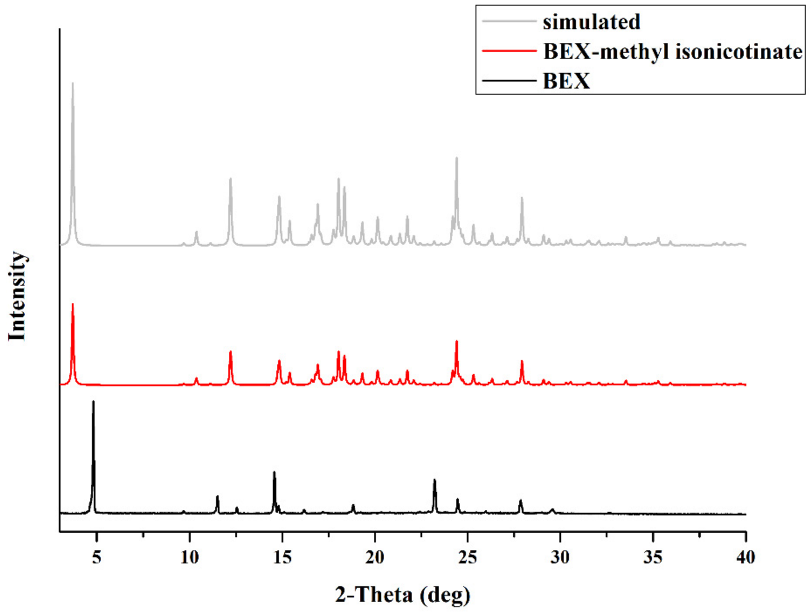

（c）

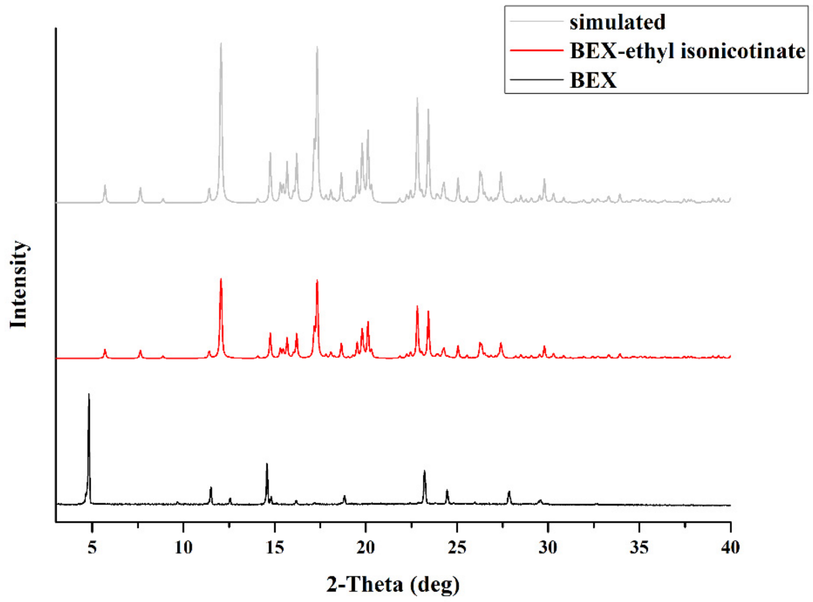

（d）

Figure S1. PXRD patterns of solid forms of BEX: (a) BEX-pyrazine, (b) BEX-2,5-dimethylpyrazine, (c) BEX- methyl isonicotinate, (d) BEX- ethyl isonicotinate.

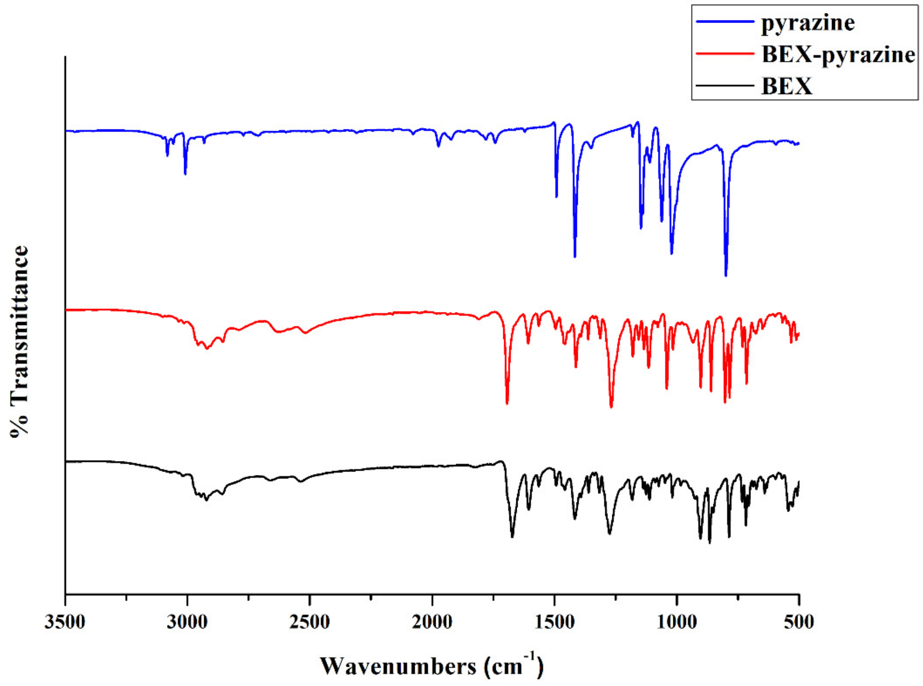
pyrazine
BEX-pyrazine
BEX
% Transmittance
3500
3000
2500
2000
1500
1000
500
Wavenumbers (cm^{-1})

（a）

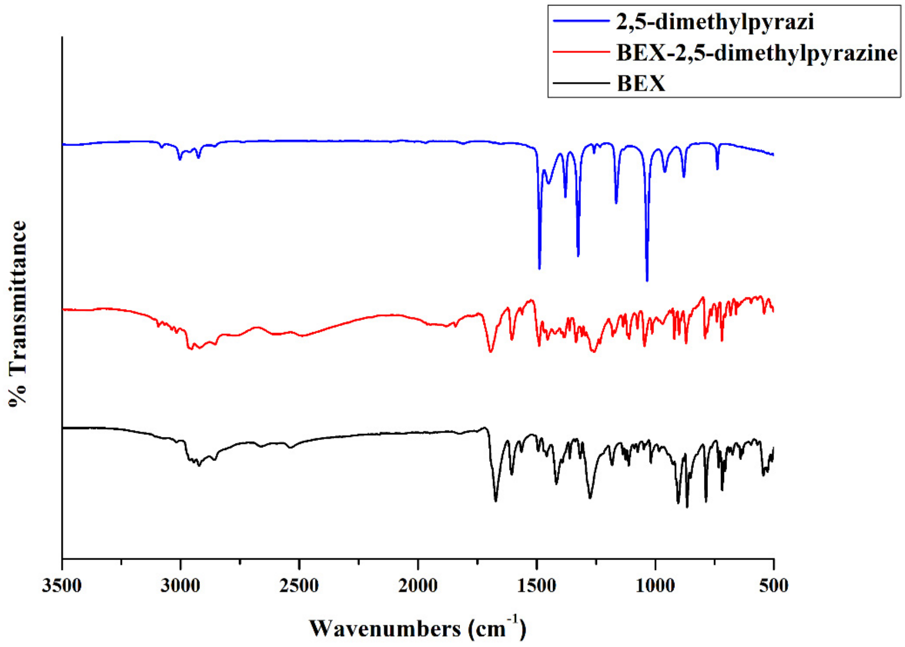
2,5-dimethylpyrazi
BEX-2,5-dimethylpyrazine
BEX
% Transmittance
3500
3000
2500
2000
1500
1000
500
Wavenumbers (cm^{-1})

（b）

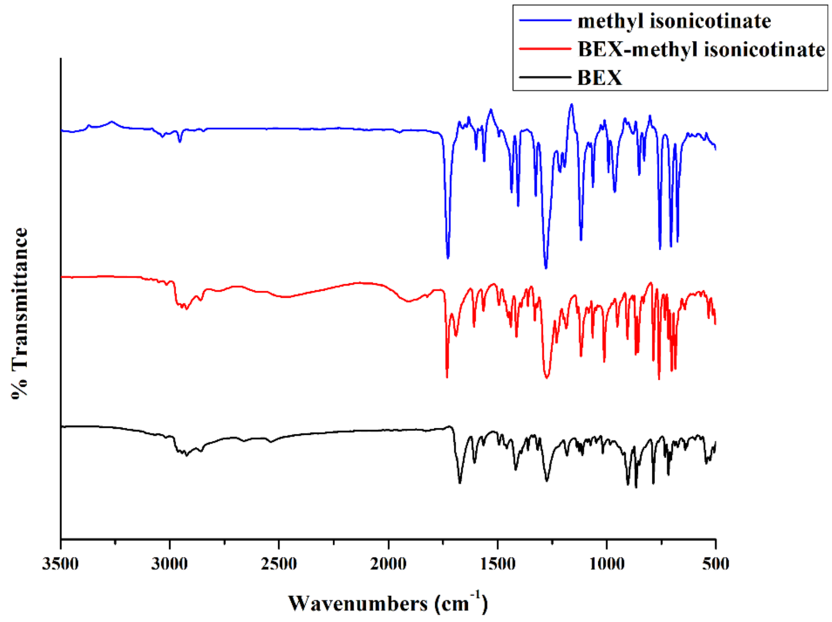

（c）

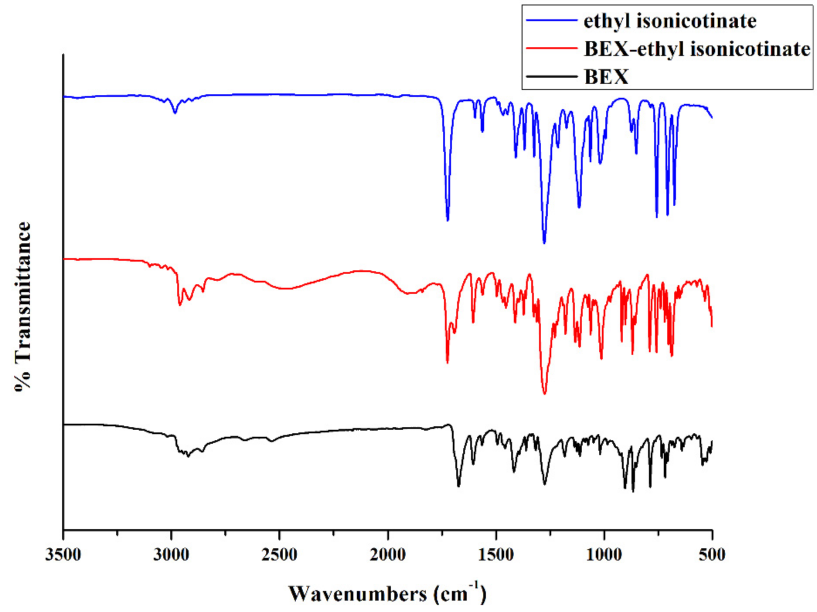

（d）

Figure S2. FTIR spectra of (a) BEX-pyrazine, (b) BEX-2,5-dimethylpyrazine, (c) BEX-methyl isonicotinate, (d) BEX- ethyl isonicotinate.

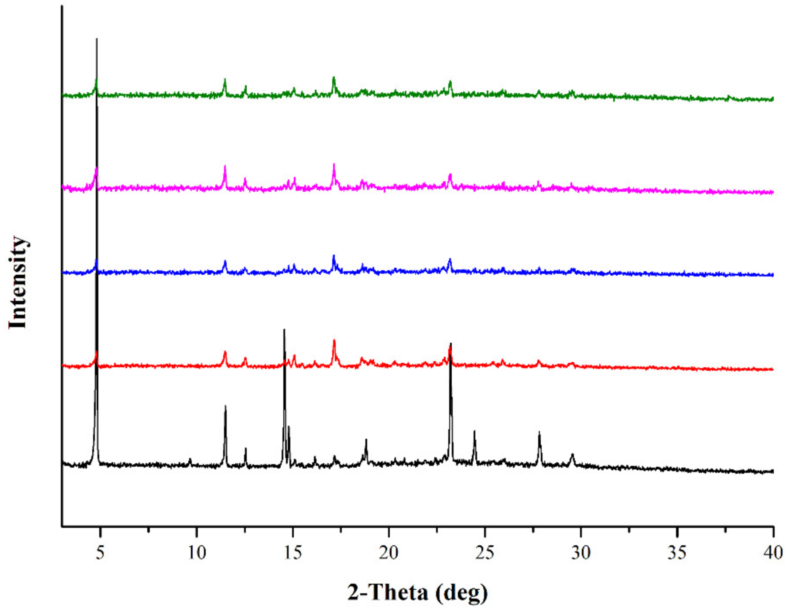
Intensity
5
10
15
20
25
30
35
40
2-Theta (deg)

(a)

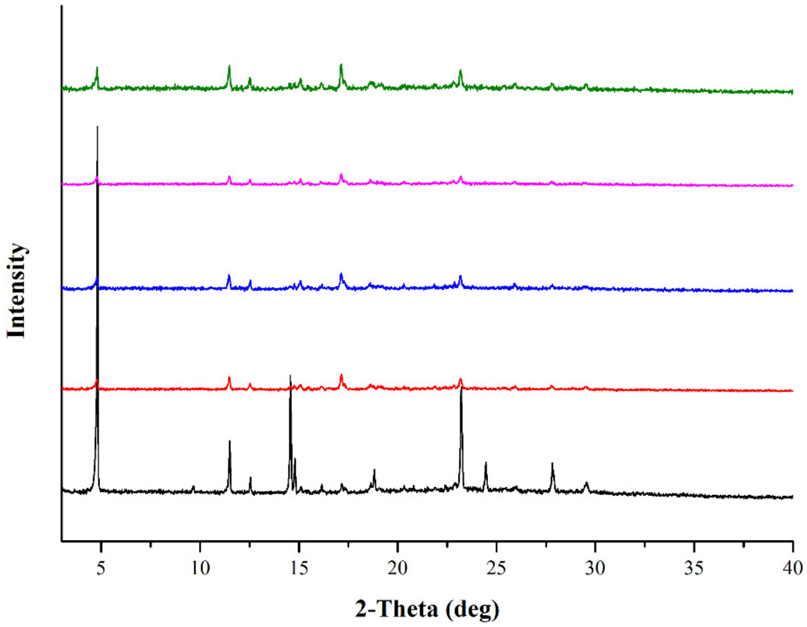
Intensity
5
10
15
20
25
30
35
40
2-Theta (deg)

(b)

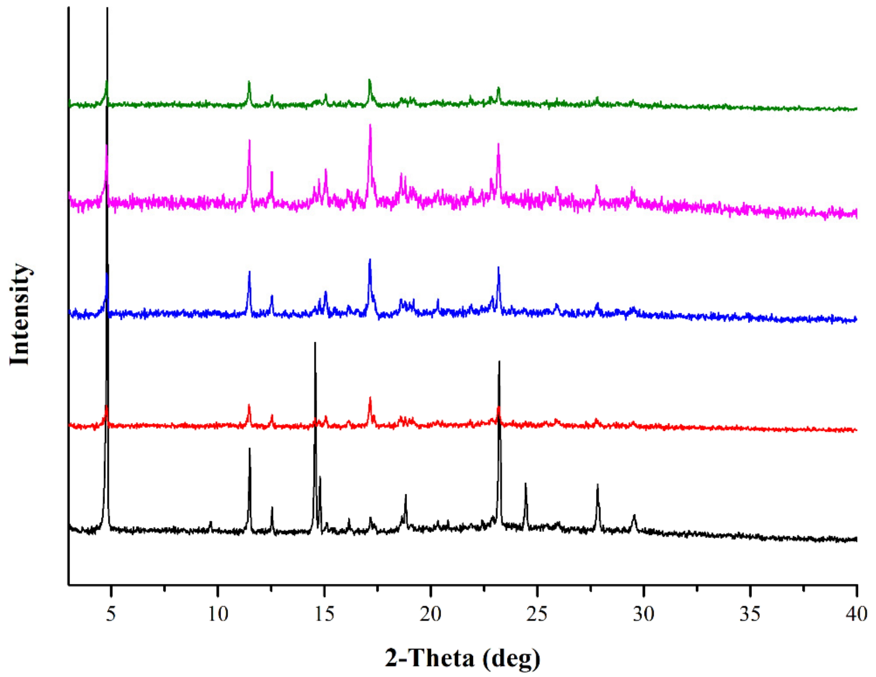

(c)

Figure S3. XRPD patterns of residues after equilibrium solubility studies. (a) pH 2.0 (b) pH 4.5 (c) pH 6.8. The XRPD pattern from top to bottom in each figure is BEX-pyrazine, BEX-2,5-dimethylpyrazine, BEX- methyl isonicotinate, BEX- ethyl isonicotinate and BEX raw materials respectively.

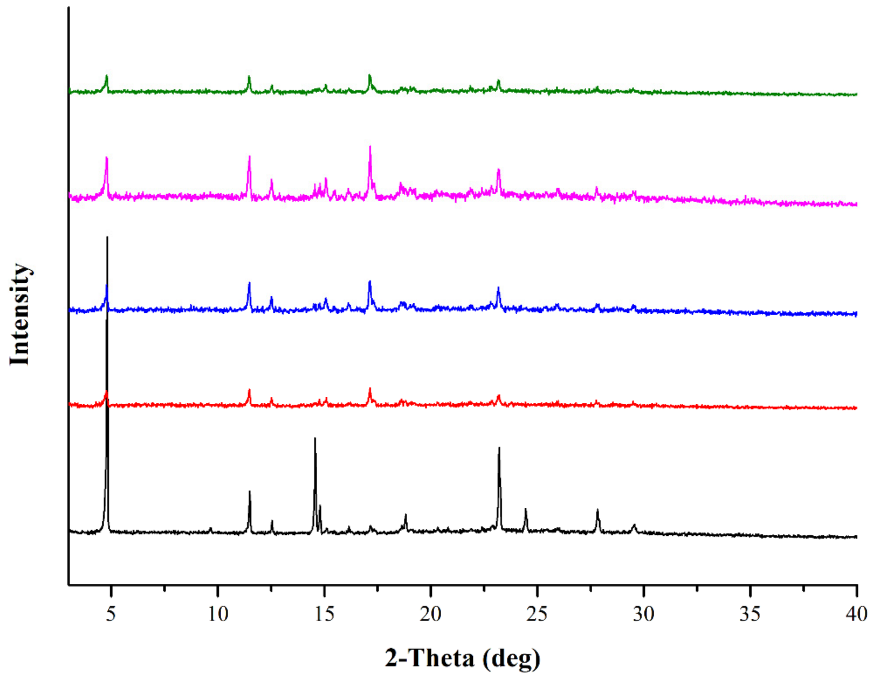

Figure S4. XRPD patterns of residues after powder dissolution. The XRPD pattern from top to bottom is BEX-pyrazine, BEX-2,5-dimethylpyrazine, BEX- methyl isonicotinate, BEX- ethyl isonicotinate and BEX raw materials respectively.

Table S1. Atom features used in CocrystalGCN.

Feature	Size	Description
Atom type	9	type of atom (ex. C, N, O, P, S, F) (one-hot)
Hybridization	5	sp, sp^2, sp^3, sp^3d, or sp^3d^2 (one-hot)
Degree	7	the number of heavy atom neighbors (one-hot)
Formal charge	7	atomic formal charge (-3 to +3, one-hot)
Implicit valence	7	the number of implicit hydrogens (0 to 6, one-hot)
Hydrogens	5	the number of neighboring hydrogens (0 to 4, one-hot)
Atomic number	1	Atomic number (integer)
Ring size	6	whether this atom belongs to a ring (3 to 8, binary)
Acid/base	2	whether this atom is acidic or basic (binary)
Hydrogen bonding	2	whether this atom is a hydrogen bond donor or acceptor (binary)
Aromaticity	1	whether this atom is part of an aromatic system (binary)

Table S2. The search spaces of hyperparamerters for CocrystalGCN.

	**Hyperparameter**	**Search spaces**
Training	Epoch	1000
	Batch size	32, 64, 128
	Patience	200, 400
	Initial learning rate	0.00015
	Loss	Binary cross-entropy
	Optimizer	SGD, Adam
Node Embedding	Number of embedding layers	1, 2
	Size of output units	128, 256, 512
Graph Convolution (Covalent)	Number of convolution layers	1, 2, 3
	Size of output units	128, 256, 512
Graph Convolution (Noncovalent)	Number of convolution layers	1, 2, 3
	Size of output units	128, 256, 512
Graph pooling	Pooling type	Sum, Max, Average
Fully connected	Number of fully connected layers	2, 3
	Size of output units	128, 256, 512
	Dropout	0.4, 0.5, 0.6
	l2 regularization	0, 0.00125, 0.0025, 0.005

Table S3. The search spaces of hyperparamerters for baseline models.

Models	Hyperparameter	Search spaces
RF	n_estimators	300, 600, 800
	max_depth	11, 21, 25, None
	max_features	auto, sqrt, log2
	criterion	gini, entropy
SVM	C	1e-3, 0.01, 0.1, 10, 100
	kernel	linear, rbf, sigmoid, poly
	gamma	0.01, 0.5, 1, auto
XGBoost	learning_rate	0.001, 0.01, 0.1, 1
	lambda	0, 1, 5
	max_depth	10, 100, 500
	n_estimators	500, 1000
DNN	solver	lbfgs, adam
	activation	tanh, relu, logistic, identity
	max_iter	300, 400, 600, 800
	hidden_layer_sizes	100, 50, 10
	learning_rate_init	0.01, 0.05, 0.1, 0.3
DeepDDS	epoch	300, 500
	batch_size	64, 128, 256
	learning_rate	0.00025, 0.0005
	dropout	0.2, 0.5
	output_dim	128, 256
	convolution layers	1, 2, 3

Table S4. Crystallographic Data of BEX cocrystals.

	BEX-pyrazine	BEX-2,5-dimethylpyrazine	BEX-methyl isonicotinate	BEX- ethyl isonicotinate
Formula	$C_{26}H_{30}NO_2$	$C_{30}H_{36}N_2O_2$	$C_{31}H_{35}NO_4$	$C_{32}H_{37}NO_4$
Crystal system	triclinic	monoclinic	triclinic	triclinic
Space group	*P-1*	*C2/c*	*P-1*	*P-1*
Temperature(K)	100	170	170	170
a (Å)	6.0104(17)	53.4719(18)	6.0219(5)	6.0211(7)
b (Å)	11.797(3)	8.3448(2)	9.2064(7)	14.894(2)
c (Å)	15.320(4)	11.6779(3)	23.840(2)	15.719(2)
α *(deg)*	90.14(5)	90	89.939(3)	81.181(4)
β *(deg)*	90.16(3)	100.189(2)	88.505(3)	85.725(4)
γ *(deg)*	90.20(6)	90	82.017(3)	86.697(4)
V ($Å^3$)	1086.3(5)	5128.6(3)	1308.45(18)	1387.6(3)
D_{Cal} (g/cm^3)	1.188	1.183	1.233	1.196
Z	2	8	2	2
λ (Mo Kα)	0.71073	0.71073	0.71073	0.71073
Independent reflns.	4430	5255	5334	5653
S	1.018	1.063	1.036	1.015
R_{int}	0.0944	0.0616	0.0527	0.0541
R_1	0.0789	0.0496	0.0580	0.0759
wR_2	0.1982	0.1669	0.1602	0.2113

Table S5. The pH values of Bex and cocrystal solutions after equilibrium solubility experiments (n = 3).

Form	pH values		
	**2.00**	**4.50**	**6.80**
BEX	2.06 ± 0.01	4.58 ± 0.01	6.82 ± 0.02
BEX-pyrazine	2.04 ± 0.02	4.60 ± 0.01	6.82 ± 0.01
BEX-2,5-dimethylpyrazine	2.07 ± 0.01	4.61 ± 0.01	6.89 ± 0.01
BEX-methyl isonicotinate	2.10 ± 0.01	4.63 ± 0.02	6.87 ± 0.01
BEX-ethyl isonicotinate	2.14 ± 0.02	4.61 ± 0.01	6.85 ± 0.03

Table S6. The pH values of Bex and cocrystal solutions after powder dissolution at pH 6.8 (n = 3).

Form	pH values
BEX	6.81 ± 0.01
BEX-pyrazine	6.82 ± 0.01
BEX-2,5-dimethylpyrazine	6.86 ± 0.02
BEX-methyl isonicotinate	6.84 ± 0.01
BEX-ethyl isonicotinate	6.86 ± 0.01
